# Adaptive CT XIGA Using LR B-Splines for Efficient Fracture Modeling

**DOI:** 10.3390/ma18050920

**Published:** 2025-02-20

**Authors:** Fei Gao, Cancan Ge, Zhuochao Tang, Jiming Gu, Rui Meng

**Affiliations:** 1 School of Management Science and Engineering, Anhui University of Technology, Ma’anshan 243032, China; 2 Key Laboratory of Multidisciplinary Management and Control of Complex Systems of Anhui Higher Education Institutes, Anhui University of Technology, Ma’anshan 243032, China; 3 AHUT Engineering Research Institute, Anhui University of Technology, Ma’anshan 243032, China

**Keywords:** adaptivity, XIGA, LR B-splines, fracture, crack propagation

## Abstract

This paper presents a novel adaptive crack-tip extended isogeometric analysis (adaptive CT XIGA) framework based on locally refined B-splines (LR B-splines) for efficient and accurate fracture modeling in two-dimensional solids. The XIGA method facilitates crack modeling without requiring the specific locations of crack faces and enables crack propagation simulation without remeshing by employing localized enrichment functions. LR B-splines, as an advanced extension of B-splines and NURBS, offer high-order continuity, precise geometric representation, and local refinement capabilities, thereby enhancing computational accuracy and efficiency. Various local mesh refinement strategies, designed based on crack and crack-tip locations, are investigated. Among these strategies, the crack-tip topological refinement strategy is adopted for local refinement in the adaptive CT XIGA framework. Stress intensity factors (SIFs) are evaluated using the contour interaction integral technique, while the maximum circumferential stress criterion is adopted to predict the crack growth direction. Numerical examples demonstrate the accuracy, efficiency, and robustness of adaptive CT XIGA. The results confirm that the proposed framework achieves superior error convergence rates and significantly reduces computational costs compared to a-posteriori-error-based adaptive XIGA methods, particularly in crack propagation simulations. These advantages establish adaptive CT XIGA as a powerful and efficient tool for addressing complex fracture problems in solid mechanics.

## 1. Introduction

Cracks in engineering structures have a significant impact on their load-bearing capacity, potentially compromising structural integrity and safety. To ensure the safe operation of such structures and to prevent catastrophic failures, gaining a comprehensive understanding of the mechanical behavior associated with fractures is essential. Numerical simulation has become an indispensable tool for investigating the mechanical behavior associated with fracture problems. Several numerical methods, such as the boundary element method (BEM) [1,2], meshfree methods (MMs) [3,4], phase-field method (PFM) [5,6], and extended finite element method (XFEM) [7,8], have been developed for crack problems. The XFEM is widely regarded as one of the most effective numerical approaches for fracture analysis. In the XFEM, standard finite element approximation is locally enriched by incorporating discontinuous displacement fields and the leading terms of the asymptotic crack-tip displacement fields, all within the framework of the partition of unity. These enrichment functions enable the XFEM to accurately capture the discontinuity across crack faces and the singularity near crack tips. As a result, the XFEM enables the entire crack to be represented independently of the computational mesh, thereby eliminating the need for remeshing during crack propagation simulations.

However, in the XFEM, geometric modeling relies on piecewise polynomial approximations, which introduce discretization errors that may reduce computational accuracy. The XFEM provides only $C^{0}$ continuity when analyzing fracture problems. To address the limitations of the XFEM, its concept has been integrated into isogeometric analysis (IGA) [9], leading to the development of extended isogeometric analysis (XIGA) [10]. The core idea of IGA is to employ the spline basis functions used in CAD for geometric representation as the shape functions in finite element analysis. This approach enables seamless integration between CAD and CAE. XIGA retains the advantages of the XFEM, including the ability to model cracks without needing to specify crack positions and to simulate crack propagation without remeshing. Additionally, it incorporates the benefits of IGA, such as higher-order continuity and precise geometric modeling. De Luycker et al. [11] employed XIGA for Mode-I crack analysis and achieved the optimal convergence rate using blending correction. Ghorashi et al. [12] were the first to use XIGA to simulate quasi-static crack propagation in 2D linear elasticity. Their results demonstrated excellent agreement between the computed SIFs and crack propagation paths in comparison to the analytical solutions and results obtained using the XFEM. Singh et al. [13] simulated three-dimensional crack problems in linear elastic materials with XIGA based on Bézier extraction. Zhong et al. [14] developed a three-dimensional rotating crack model using the XIGA method to perform vibration analysis of blades containing inclined single and multiple cracks. Khatir et al. [15] studied single- and multiple-crack identification through POD-RBF XIGA and the Jaya algorithm. Extended isogeometric analysis has also found applications in various fields, including thermo-mechanical fracture [16,17], fatigue crack growth [18,19,20], and orthotropic fracture materials [21,22,23]. Other notable applications include cohesive fracture [24,25], cracks in piezoelectric materials [26,27,28], T-stress evaluation [29,30], and optimization [31,32].

XIGA has found extensive applications in the study of fracture problems in plate and shell structures, attributed to its inherent high-order continuity. Nguyen-Thanh et al. [33] proposed an extended isogeometric analysis formulation to evaluate through-the-thickness cracks in thin shell structures based on Kirchhoff–Love theory. Nguyen et al. [34] investigated the vibration behavior of functionally graded microplates containing cracks. The study employed strain gradient theory with a single material length scale parameter and an additional micro-inertia term in conjunction with XIGA. Singh et al. [35] utilized XIGA to investigate the behavior of cracked functionally graded material (FGM) plates based on a generalized higher-order shear deformation theory. Yang et al. [36] integrated XIGA with the finite cell method to study the vibration and buckling behavior of FGM plates containing multiple internal cracks and cutouts under combined thermal and mechanical loads. Singh et al. [37] presented an extended isogeometric analysis for cracked functionally graded magneto-electro-elastic materials. Yadav et al. [38] conducted a pioneering study on the fracture behavior of carbon-nanotube-reinforced functionally graded structures with discontinuities under thermo-mechanical loading utilizing XIGA. Kumar et al. [39] conducted a comprehensive fracture analysis of edge-cracked porous functionally graded structures, considering various porosity distribution patterns under mechanical loading.

To accurately simulate fracture problems, fine elements are required in the vicinity of the crack. While using fine elements throughout the entire domain would ensure high computational accuracy, it would also result in excessively high computational costs. To improve computational efficiency without compromising accuracy, fine elements are employed near the crack region, while coarser elements are utilized in regions farther from the crack. Adaptive XIGA or multi-scale XIGA has been successfully applied to address fracture problems under such scenarios. Ghorashi et al. [40] were the first to apply multi-scale XIGA for the fracture analysis of orthotropic cracked media. Their approach utilized T-splines, which enable efficient local refinements to enhance computational accuracy and efficiency. Nguyen-Thanh et al. [41] utilized adaptive XIGA based on PHT-splines to investigate crack propagation in the vicinity of inclusions. Gu et al. [42,43,44] employed adaptive XIGA utilizing LR B-splines to model fracture problems and simulate crack propagation in both 2D isotropic and orthotropic materials. Yuan et al. [45] studied crack propagation in complex Mindlin–Reissner plates by developing an adaptive multi-patch XIGA according to LR NURBS and Nitsche’s method. The adaptive XIGA method, based on LR B-splines or LR NURBS, has also been applied to simulate arbitrary holes in orthotropic media [46], perform upper-bound limit analysis [47,48], and model cracked composite FG Mindlin–Reissner plates [49]. Schmidt et al. [50] developed the adaptive XIGA framework to predict the behavior of multi-material multiphysics problems with complex geometries, employing locally refined discretizations based on hierarchical B-splines. Jiang et al. [51] proposed adaptive XIGA with the strong imposition of essential boundary conditions for linear elastic fracture problems based on B++ splines. Qiu et al. [52] presented an adaptive XIGA method based on PHT-splines for solving phase-field fracture mechanics problems.

In our previous works [42,43,44], we developed an adaptive XIGA method based on LR B-splines for fracture analysis, focusing on the computation of SIFs and crack propagation. Adaptive local mesh refinement was achieved using a posteriori error estimation, in which the error was computed through recovery techniques. We observed that adaptive XIGA based on a posteriori error estimation facilitates fine refinement near the crack. In contrast, coarse-scale mesh refinement is applied in regions that are farther from the crack. This approach achieves high accuracy with reduced degrees of freedom, yielding an error convergence rate that is superior to global refinement. However, the application of a-posteriori-error-based adaptive XIGA to fracture problems, particularly crack propagation, entails significant computational costs. The primary reason lies in the computational demands of a-posteriori-error-based adaptive XIGA with local mesh refinement for calculating SIFs. This process requires multiple displacement solutions, along with stress or strain recovery, where each recovery step incurs a computational cost equivalent to that of solving a displacement solution. Furthermore, our previous studies revealed that adaptive local refinement predominantly occurs near the crack tip during crack propagation.

In this paper, we propose an adaptive CT XIGA framework based on LR B-splines for solving fracture problems in 2D elastic solids, including the calculation of stress intensity factors and the simulation of crack propagation. LR B-splines, as an extension of B-splines and NURBS, inherit precise geometric modeling capabilities and high-order continuity while enabling local mesh refinement. The adaptive CT XIGA framework incorporates the generalized Heaviside function and crack-tip enrichment functions to effectively capture the discontinuities and singularities induced by cracks. To identify regions for local refinement, we propose multiple mesh refinement strategies based on the positions of cracks and crack tips. We compare the error convergence rates and computational times of each local refinement strategy with those of both a-posteriori-error-based mesh refinement and uniform global mesh refinement. Based on these comparisons, we identify the most effective local refinement strategy, termed the crack-tip topological refinement strategy. The adaptive XIGA framework employing this strategy provides the following advantages: (i) crack modeling is independent of the crack position, and crack propagation can be simulated without remeshing. (ii) Compared to the XFEM, the proposed method achieves precise geometric modeling with higher accuracy and convergence rates. (iii) With local mesh refinement near the crack tip, the proposed method achieves higher accuracy at the same degrees of freedom compared to traditional XIGA. (iv) The crack-tip topological refinement strategy simplifies the implementation of local mesh refinement compared to a-posteriori-error-based methods. (v) Compared to a-posteriori-error-based adaptive XIGA, the proposed approach achieves higher error convergence rates while significantly reducing computational time, particularly in crack propagation simulations. The advantages of the proposed method are validated through numerical examples.

The structure of the paper is as follows: Section 2 begins with a brief overview of LR B-splines, B-splines, and NURBS. This is followed by the formulation of the XIGA approach applied to crack problems. Then, the computational formulas for SIFs and crack propagation are outlined. In Section 3, several numerical examples are provided to demonstrate the effectiveness of the proposed method. Finally, the conclusions of this study are presented in Section 4.

## 2. Adaptive XIGA Based on LR B-Splines for Cracked Media

### 2.1. LR B-Spline-Based Local Refinement

B-splines and non-uniform rational B-splines (NURBS), owing to their properties, including higher-order continuity and precise geometric representation, are widely used as basis functions in IGA and XIGA. However, their tensor-product structure poses challenges for performing local mesh refinement. LR B-splines were introduced by Dokken et al. [53] to overcome certain limitations associated with NURBS and B-splines. LR B-splines are a generalization of NURBS and B-splines, retaining their advantageous properties while enabling local refinement. The B-spline basis functions are defined using a knot vector $\Xi =\left\{\xi_{1},\xi_{2},\cdots ,\xi_{n+p+1}\right\}$, where $\xi_{i}\in R$ are non-decreasing coordinates in the parameter space. Here, *n* denotes the number of basis functions, and *p* represents their degree. The *i*th B-spline basis function $N_{i,p}$ of degree *p* is recursively defined as follows:

(1) $$N_{i,0}\left(\xi \right)=\left\{\begin{array}{cc} 1 & \mathrm{if}\;\xi_{i}\leq \xi <\xi_{i+1}\\ 0 & \mathrm{otherwise} \end{array}\right.,\;p=0,$$

(2) $$N_{i,p}\left(\xi \right)=\frac{\xi -\xi_{i}}{\xi_{i+p}-\xi_{i}}N_{i,p-1}\left(\xi \right)+\frac{\xi_{i+p+1}-\xi }{\xi_{i+p+1}-\xi_{i+1}}N_{i+1,p-1}\left(\xi \right),\;p\geq 1.$$

NURBS are derived from B-splines and associated weights, enabling the exact representation of a wide variety of complex geometries. Given a B-spline basis function $N_{i,p}\left(\xi \right)$ and weight $w_{i}\in R$, a univariate NURBS of degree *p* is constituted by

(3) $$R_{i,p}\left(\xi \right)=\frac{N_{i,p}\left(\xi \right)w_{i}}{\sum_{j=1}^{n}N_{j,p}\left(\xi \right)w_{j}}.$$

A bivariate NURBS is expressed by

(4) $$R_{i,j}^{p,q}\left(\xi ,\eta \right)=\frac{N_{i,p}\left(\xi \right)M_{j,q}\left(\eta \right)w_{i,j}}{\sum_{k=1}^{n}\sum_{l=1}^{m}N_{k,p}\left(\xi \right)M_{l,q}\left(\eta \right)w_{k,l}},$$

where $w_{i,j}\in R$ represents the weight; $N_{i,p}\left(\xi \right)$ is the B-spline basis function of degree *p* in the ξ direction, which is defined on the knot vector $\Xi =\left\{\xi_{1},\xi_{2},\cdots ,\xi_{n+p+1}\right\}$; $M_{j,q}\left(\eta \right)$ is the B-spline basis function of degree *q* in the η direction, which is defined on the knot vector $H=\left\{\eta_{1},\eta_{2},\cdots ,\eta_{m+q+1}\right\}$.

An LR B-spline basis function of degree *p* in ξ direction is a weighted B-spline basis function with minimum support on an LR mesh and is expressed as follows:

(5) $$B_{\Xi_{i}}^{\gamma }\left(\xi \right)=\gamma^{i}B_{\Xi_{i}}\left(\xi \right),$$

where $\gamma^{i}\in \left(0,1\right]$ is a scalar weight factor ensuring that LR B-splines maintain the partition of unity property; $B_{\Xi_{i}}\left(\xi \right)=N_{i,p}\left(\xi \right)$ denotes a B-spline basis function defined on a local knot vector $\Xi_{i}$. The set of local knot vectors $\Xi_{i}$ with p+2 knots, extracted from the global knot vector $\Xi =\left\{\xi_{1},\xi_{2},\cdots ,\xi_{n+p+1}\right\}$, is defined as

(6) $$\Xi_{i}={\left\{\xi_{i+j}\right\}}_{j=0}^{p+1},\;i=1,\cdots ,n.$$

A bivariate LR B-spline basis function of degree *p* in ξ direction and degree *q* in η direction is defined as follows:

(7) $$B_{\Xi_{k},H_{k}}^{\gamma_{k}}\left(\xi ,\eta \right)=\gamma^{k}B_{\Xi_{i}}\left(\xi \right)B_{H_{j}}\left(\eta \right)=\gamma^{k}N_{i,p}\left(\xi \right)M_{j,q}\left(\eta \right).$$

Similar to the construction of NURBS using B-splines, LR NURBS can be constructed using LR B-splines, expressed as follows:

(8) $$R_{\Xi_{k},H_{k}}^{\gamma_{k}}\left(\xi ,\eta \right)=\frac{B_{\Xi_{k},H_{k}}^{\gamma_{k}}\left(\xi ,\eta \right)w_{k}}{\sum_{d=1}^{n_{cp}}B_{\Xi_{d},H_{d}}^{\gamma_{d}}\left(\xi ,\eta \right)w_{d}},$$

where $n_{cp}$ denotes the number of control points.

Since LR NURBS can be formulated from LR B-splines and LR B-splines are a special case of LR NURBS where all weights $\gamma_{k}$ are equal to 1, LR B-splines will be used to represent both LR B-splines and LR NURBS throughout the remainder of this text. For further details on LR B-splines and LR NURBS, the reader is referred to Ref. [53].

### 2.2. XIGA Formulation for Cracked Media

**Figure 1:**
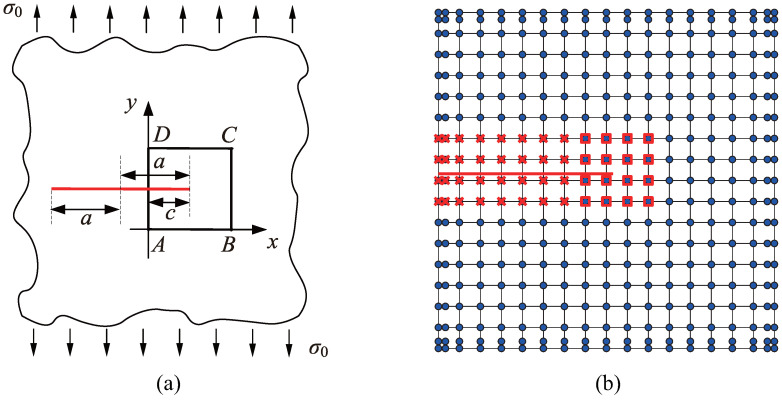
(**a**) Schematic representation of an infinite plate with a central crack under remote uniform tensile loading. (**b**) Initial computational mesh and control points. The blue dots represent the control points, while the red line depicts the crack. The red square symbols mark control points enriched with crack-tip enrichment functions, whereas the red cross symbols denote control points enriched with the Heaviside function.

The XIGA is formed by incorporating the concepts of the XFEM into conventional IGA, enabling the simulation of cracks without considering the locations of cracks, and modeling crack propagation without remeshing. In XIGA, the isogeometric approximation of the displacement field is locally enriched to capture the discontinuity across crack faces and the singularity near crack tips. The XIGA approximation for a 2D crack problem is mathematically expressed as follows [42]:

(9) $$u^{h}\left(\xi \right)=\sum_{i\in N^{std}}R_{i}\left(\xi \right)u_{i}+\sum_{j\in N^{cf}}R_{j}\left(\xi \right)H_{j}\left(\xi \right)d_{j}+\sum_{k\in N^{ct}}R_{k}\left(\xi \right)\sum_{\alpha =1}^{4}Q_{k}^{\alpha }\left(\xi \right)c_{k}^{\alpha },$$

with

(10) $$\begin{array}{cc} \; & H_{j}\left(\xi \right)=H\left(\xi \right)-H\left(\xi_{j}\right), \end{array}$$

(11) $$\begin{array}{cc} \; & Q_{k}^{\alpha }\left(\xi \right)=Q_{\alpha }\left(\xi \right)-Q_{\alpha }\left(\xi_{k}\right), \end{array}$$

(12) $$\begin{array}{cc} \; & \left\{Q_{1},Q_{2},Q_{3},Q_{4}\right\}=\left\{\sqrt{r}\sin\frac{\theta }{2},\sqrt{r}\cos\frac{\theta }{2},\sqrt{r}\sin\theta \sin\frac{\theta }{2},\sqrt{r}\sin\theta \cos\frac{\theta }{2}\right\}, \end{array}$$

where H(ξ) is the generalized Heaviside function employed to capture the discontinuity across crack faces, which takes the value +1 on one side of the crack and −1 on the opposite side; $Q_{\alpha }\left(\xi \right)$ denotes crack-tip enrichment functions to simulate singularity in the vicinity of crack tips; $R_{i}$, $R_{j}$, and $R_{k}$ represent the LR B-spline basis functions; $u_{i}$, $d_{j}$, and $c_{k}^{\alpha }$ are the displacement control variables related to the standard IGA, the additional degrees of freedom (DOFs) associated with modeling crack faces, and the additional DOFs associated with crack tips, respectively; $N^{std}$, $N^{cf}$, and $N^{ct}$ denote the sets of LR B-splines in the whole discretization domain, supporting the elements containing crack faces (excluding crack tips), and whose support domain contains crack tips, respectively. The elements containing crack tips are referred to as crack-tip elements, while the elements containing crack faces (excluding crack tips) are referred to as crack-face elements. The control points whose corresponding basis functions are enriched by the Heaviside function and crack-tip enrichment functions are referred to as crack-face-enriched control points and crack-tip-enriched control points, respectively. These are illustrated in Figure 1b.

The displacement, strain, and stress at any point are written as follows:

(13) $$\begin{array}{c} u^{h}=RU,\;\varepsilon^{h}=BU,\;\sigma^{h}=DBU, \end{array}$$

where R stands for the basis function matrix and B is the strain–displacement matrix. For the details of R and B, the reader is referred to Ref. [42].

### 2.3. SIF Evaluation and Direction of Crack Growth

Fracture criteria and crack growth direction are critical topics in crack propagation research. Various criteria are employed to determine the direction of crack propagation. Considering efficiency and ease of implementation, the maximum circumferential stress criterion [54] is commonly used to predict the crack propagation direction. According to the maximum circumferential stress criterion, the crack growth direction $\theta_{c}$ is written as

(14) $$\theta_{c}=2\arctan\left(\frac{1}{4}\left[\frac{K_{I}}{K_{II}}\pm \sqrt{{\left(\frac{K_{I}}{K_{II}}\right)}^{2}+8}\right]\right).$$

In Equation (14), $K_{I}$ and $K_{II}$ denote the Mode-I and Mode-II SIFs, respectively. The interaction integral technique is applied to compute the mixed-mode SIFs. The formulation for mixed-mode SIFs is provided by [51]

(15) $$\left[\begin{array}{c} K_{I}^{\left(1\right)}\\ K_{II}^{\left(1\right)} \end{array}\right]=\frac{E^{*}}{2}\left[\begin{array}{c} I^{\left(1,\mathrm{mode}\;I\right)}\\ I^{\left(1,\mathrm{mode}\;\mathrm{II}\right)} \end{array}\right],$$

where

(16) $$I^{\left(1,2\right)}=\int_{A}\left[\sigma_{ij}^{\left(1\right)}\frac{\partial u_{i}^{\left(2\right)}}{\partial x_{1}}+\sigma_{ij}^{\left(2\right)}\frac{\partial u_{i}^{\left(1\right)}}{\partial x_{1}}-W^{\left(1,2\right)}\delta_{1j}\right]\frac{\partial q}{\partial x_{j}}dA,$$

and

(17) $$E^{*}=\left\{\begin{array}{c} E\;\;\;\;\;\;\;\;\;\;\;\;\;\mathrm{plane}\;\mathrm{stress}\\ \frac{E}{1-\nu^{2}}\;\;\;\;\;\;\mathrm{plane}\;\mathrm{strain} \end{array}\right.$$

In Equation (16), $\left(\sigma_{ij}^{\left(1\right)},\varepsilon_{ij}^{\left(1\right)},u_{i}^{\left(1\right)}\right)$ and $\left(\sigma_{ij}^{\left(2\right)},\varepsilon_{ij}^{\left(2\right)},u_{i}^{\left(2\right)}\right)$ represent the actual and auxiliary states, respectively. *A* denotes the area enclosed by the contour. The function *q* is defined as 1 within the integration area *A* and 0 outside. $W^{1,2}=\left(\sigma_{ij}^{\left(1\right)}\varepsilon_{ij}^{\left(2\right)}+\sigma_{ij}^{\left(2\right)}\varepsilon_{ij}^{\left(1\right)}\right)/2$ represents the interaction strain energy. In Equation (17), *E* and ν are the elastic modulus and Poisson’s ratio, respectively.

## 3. Numerical Examples

To validate the accuracy and efficiency of the methodology proposed herein, six numerical examples are presented in this section. The first three examples focus on the calculation of SIFs, while the subsequent three examples simulate crack propagation. The validity and effectiveness of the adaptive extended isogeometric analysis, based on crack-tip locations for addressing fracture issues, are confirmed by comparing the computed results with exact or reference solutions. This study investigates quasi-static crack propagation under the assumption that all cracks are assumed to propagate freely. Unless otherwise specified, all examples in this section adopt LR B-spline basis functions with third-order polynomial directions (p=q=3), consistent with Refs. [42,43]. The red line represents the crack in the figures below. Plane strain conditions are assumed. Convergence curves of error are plotted on a natural logarithmic scale lg(), and seconds are used as the time unit for calculations. The structured mesh refinement strategy [55] is employed to locally refine the LR mesh. The numerical integration is conducted in a manner consistent with previous works [42,43]. The adaptive extended isogeometric analysis program in this study is implemented using a hybrid MATLAB R2023b–C++ 11.4.0 programming approach. All simulations are performed on a single workstation equipped with a 14th-generation Intel Core i9 processor (3.2 GHz) and 64 GB of memory. To ensure optimal performance and accuracy, the workstation is exclusively allocated for running the program, with no other tasks handled during execution.

### 3.1. Infinite Plate with a Center Crack

An infinite plate with a central straight crack, subjected to a unidirectional uniform tensile force of $\sigma_{0}=10^{4}$ units, is illustrated in Figure 1a. The crack length is defined as 2a=200 units. To effectively deal with the infinite domain, a square region ABCD enclosing the crack is selected for analysis. The square has a side length of 10 units, with the crack having a length of c=5.2 units. A coordinate system is established with vertex *A* as the origin, where the direction AB serves as the x-axis and AD serves as the y-axis. The coordinates of the crack tip are specified as (5.2,5.2). The material properties include elastic modulus of $E=10^{7}$ units and Poisson’s ratio ν=0.3. Using a local polar coordinate system centered at the crack tip, the analytical solutions for the displacement and stress fields are provided as follows [42]:

(18) $$\begin{array}{cc} u_{x}\left(r,\theta \right) & =\frac{2\left(1+\nu \right)}{\sqrt{2\pi }}\frac{K_{I}}{E}\sqrt{r}\cos\frac{\theta }{2}\left(2-2\nu -\cos^{2}\frac{\theta }{2}\right),\\ u_{y}\left(r,\theta \right) & =\frac{2\left(1+\nu \right)}{\sqrt{2\pi }}\frac{K_{I}}{E}\sqrt{r}\sin\frac{\theta }{2}\left(2-2\nu -\cos^{2}\frac{\theta }{2}\right), \end{array}$$

(19) $$\begin{array}{cc} \sigma_{xx}\left(r,\theta \right) & =\frac{K_{I}}{\sqrt{2\pi r}}\cos\frac{\theta }{2}\left(1-\sin\frac{\theta }{2}\sin\frac{3\theta }{2}\right),\\ \sigma_{yy}\left(r,\theta \right) & =\frac{K_{I}}{\sqrt{2\pi r}}\cos\frac{\theta }{2}\left(1+\sin\frac{\theta }{2}\sin\frac{3\theta }{2}\right),\\ \sigma_{xy}\left(r,\theta \right) & =\frac{K_{I}}{\sqrt{2\pi r}}\sin\frac{\theta }{2}\cos\frac{\theta }{2}\cos\frac{3\theta }{2}, \end{array}$$

where (r,θ) denotes the polar coordinate system centered at the crack tip; $K_{I}=\sigma_{0}\sqrt{\pi a}$ denotes the Model-I SIF.

In the numerical analysis, exact displacement boundary conditions are imposed on the top, bottom, and right edges of the square ABCD in accordance with the analytical solution for displacements outlined in Equation (18). Exact stress boundary conditions are applied to the left edge of the square ABCD according to the analytical stress solution presented in Equation (19). The initial computational mesh consists of 16×16 elements, as illustrated in Figure 1b.

Firstly, the localized refinement regions around the crack are investigated. As the structured mesh refinement strategy is employed, the focus is on determining which LR B-spline basis functions require refinement. Two main categories are considered as follows:

1. Refinement is applied only to the localized region near the crack tip, ignoring the crack-face region.
2. Refinement is applied both to the localized region near the crack tip and the area surrounding the crack face.

For mesh refinement in the localized region around the crack tip, two scenarios are considered as follows:

- Refinement is applied to the LR B-spline basis functions supporting the crack-tip element, referred to as the crack-tip topological refinement strategy.
- A refinement radius $R_{r}=n_{r}\sqrt{S_{tip}}$, where $n_{r}\in N_{+}$ and $S_{tip}$ represent the area of the crack-tip element. Refinement is applied to the LR B-spline basis functions supporting elements within the circular region of radius $R_{r}$ centered at the crack tip. This approach is referred to as the crack-tip geometric refinement strategy. The crack-tip topological refinement strategy can be considered a special case of the crack-tip geometric refinement strategy where $n_{r}=1$. This study specifically examines cases where $n_{r}=2$ or 3. Regions with $n_{r}>3$, being farther from the crack tip, are excluded from refinement.

**Figure 2:**
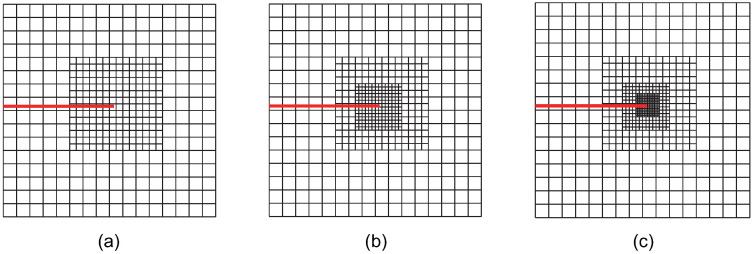
Meshes at the first (**a**), second (**b**), and third (**c**) refinement steps obtained by adaptive CT XIGA.

**Figure 3:**
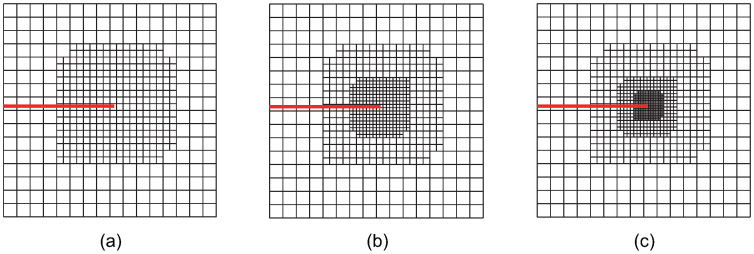
Meshes at the first (**a**), second (**b**), and third (**c**) refinement steps obtained by adaptive CT2 XIGA.

**Figure 4:**
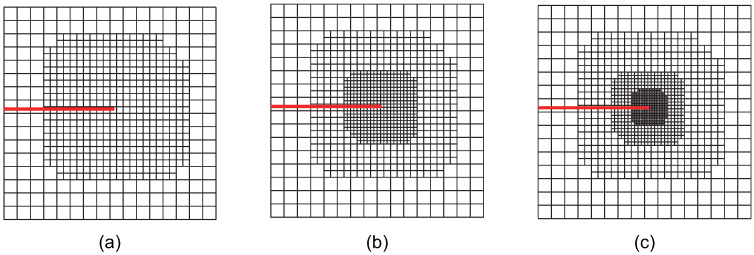
Meshes at the first (**a**), second (**b**), and third (**c**) refinement steps obtained by adaptive CT3 XIGA.

**Figure 5:**
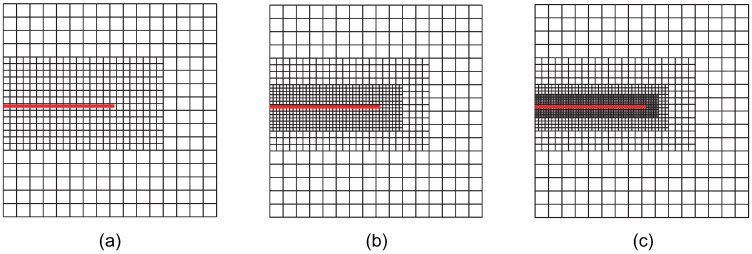
Meshes at the first (**a**), second (**b**), and third (**c**) refinement steps obtained by adaptive CTCF XIGA.

**Figure 6:**
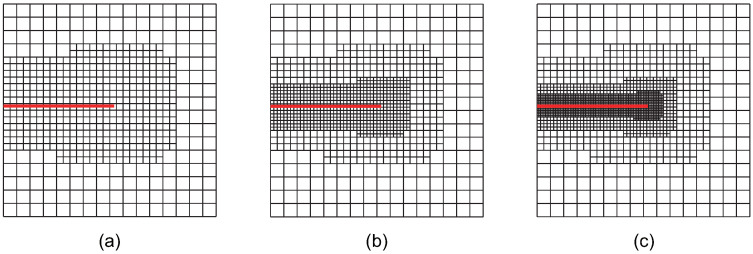
Meshes at the first (**a**), second (**b**), and third (**c**) refinement steps obtained by adaptive CT2CF XIGA.

**Figure 7:**
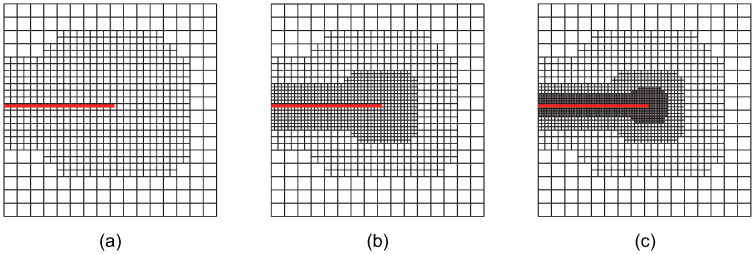
Meshes at the first (**a**), second (**b**), and third (**c**) refinement steps obtained by adaptive CT3CF XIGA.

For the crack-face region, refinement is utilized for all non-zero LR B-spline basis functions supporting the crack-face elements. The various refinement strategies are classified and analyzed. Using these refinement strategies in accordance with the structured mesh refinement strategy, the first three steps of adaptive local refinement meshes are obtained, as shown in Figure 2, Figure 3, Figure 4, Figure 5, Figure 6 and Figure 7. These strategies are classified into six main cases:

Case 1: The crack-tip topological refinement strategy. Adaptive XIGA with this strategy is referred to as adaptive crack-tip XIGA (adaptive CT XIGA), as shown in Figure 2.

Case 2: The crack-tip geometric refinement strategy with $n_{r}=2$. Adaptive XIGA with this strategy is referred to as adaptive crack-tip 2 XIGA (adaptive CT2 XIGA), as shown in Figure 3.

Case 3: The crack-tip geometric refinement strategy with $n_{r}=3$. Adaptive XIGA with this strategy is referred to as adaptive crack-tip 3 XIGA (adaptive CT3 XIGA), as shown in Figure 4.

Case 4: A combined strategy involving crack-tip topological refinement and crack-face refinement. Adaptive XIGA with this strategy is referred to as adaptive crack-tip and crack-face XIGA (adaptive CTCF XIGA), as shown in Figure 5.

Case 5: A combined strategy involving crack-tip geometric refinement with $n_{r}=2$ and crack-face refinement. Adaptive XIGA with this strategy is referred to as adaptive crack-tip 2 and crack-face XIGA (adaptive CT2CF XIGA), as shown in Figure 6.

Case 6: A combined strategy involving crack-tip geometric refinement with $n_{r}=3$ and crack-face refinement. Adaptive XIGA with this strategy is referred to as adaptive crack-tip 3 and crack-face XIGA (adaptive CT3CF XIGA), as shown in Figure 7.

Figure 2, Figure 3, Figure 4, Figure 5, Figure 6 and Figure 7 show that, for all refinement strategies, both the mesh sizes around the crack tip and the refinement zones decrease as the number of refinement steps increases. For the crack-tip geometric refinement strategy, the refinement zone expands as $n_{r}$ increases. Among all the strategies, the crack-tip topological refinement strategy yields the smallest refinement zone.

**Figure 8:**
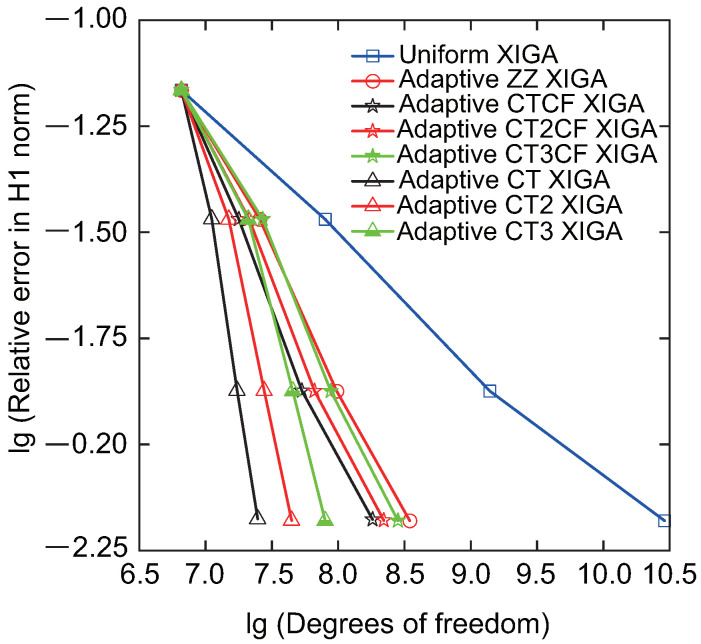
Convergence of the relative error in the H1 norm as a function of the number of DOFs for adaptive XIGA using various refinement strategies.

**Figure 9:**
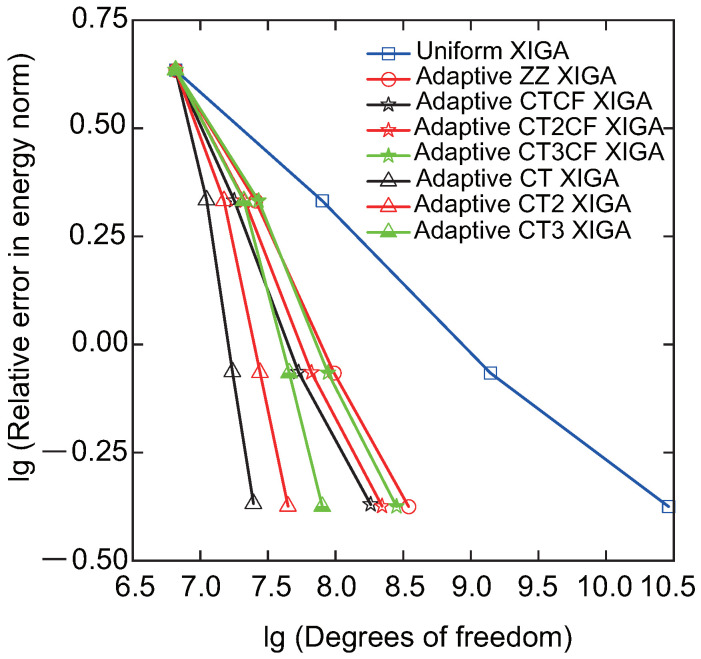
Convergence of the relative error in the energy norm as a function of the number of DOFs for adaptive XIGA using various refinement strategies.

Then, the convergence rate of relative errors in the H1 and energy norms, as a function of the number of DOFs, is analyzed for adaptive XIGA under various refinement strategies, as displayed in Figure 8 and Figure 9. The formulations for the relative errors in the H1 and energy norms are provided in Ref. [42]. For comparison, the results of the XIGA with adaptive local refinement based on a posteriori error estimation and uniform global refinement [42] are also presented. The adaptive XIGA approach using local refinement based on a posteriori error estimation with the recovery technique proposed by Zienkiewicz and Zhu [56] is called adaptive ZZ XIGA, while the XIGA with uniform global refinement is called uniform XIGA. All mesh refinement strategies exhibit a decrease in relative errors as the number of DOFs increases, converging to smaller values after three mesh refinement steps. Notably, the natural logarithm values for the relative errors in H1 norm and energy norm stabilize around −2.2 and −0.37, respectively, while their actual values approach 0.111% and 0.691%. Such minimal errors underscore the accuracy and efficiency of adaptive XIGA based on LR B-splines under various refinement strategies for addressing fracture problems.

Among the various strategies examined, the error convergence rate of the crack-tip mesh refinement strategy is generally superior to that of the combined strategy involving both crack-tip and crack surface mesh refinement. It is also faster than that of uniform global refinement and a-posteriori-error-based adaptive mesh refinement strategies, as shown in Figure 8 and Figure 9. Furthermore, the crack-tip topological mesh refinement strategy exhibits the highest error convergence rate among all the refinement strategies. The adaptive XIGA method adopts the topological mesh refinement strategy, performing two and three local mesh refinement steps, respectively. This approach achieves relative errors of 0.0221% and 0.0056% for Mode-I SIF, demonstrating its high accuracy and efficiency.

**Figure 10:**
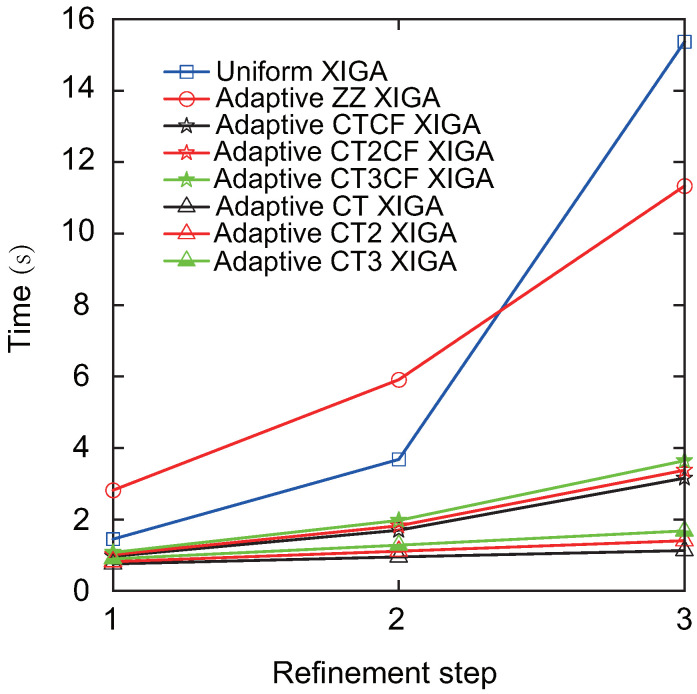
Comparison of computation times of adaptive XIGA using various refinement strategies.

Finally, the computation time required to determine the Mode-I SIF at different refinement steps derived from adaptive XIGA with various refinement strategies is discussed, as illustrated in Figure 10. For all the refinement strategies, the computation time increases as the number of refinement steps increases. However, the computation time growth rate of adaptive XIGA with six refinement strategies proposed in this paper is significantly lower than that of uniform XIGA and adaptive ZZ XIGA. In particular, adaptive CT XIGA shows the slowest growth rate in computation time and has the shortest computation time at the 1st, 2nd, and 3rd refinement steps among all the strategies. The computation times for adaptive CT XIGA to calculate the Mode-I SIF at the 1st, 2nd, and 3rd refinement steps are 0.75 s, 0.95 s, and 1.13 s, respectively. It is noteworthy that the longer computation time for uniform XIGA results from the increased number of mesh elements and degrees of freedom caused by global uniform refinement. Although adaptive ZZ XIGA can reduce the number of elements and degrees of freedom to some extent, the computational cost remains high due to multiple recovery steps and displacement field calculations [42]. The computation times for uniform XIGA and adaptive ZZ XIGA to calculate the SIF at the 3rd refinement step are 15.36 s and 11.33 s, respectively, which are 11.33 times and 10.04 times longer than that of adaptive CT XIGA. Due to its high convergence rate and low computational cost, adaptive CT XIGA is adopted for the remaining numerical examples.

### 3.2. Circular Plate with a Central Crack

**Figure 11:**
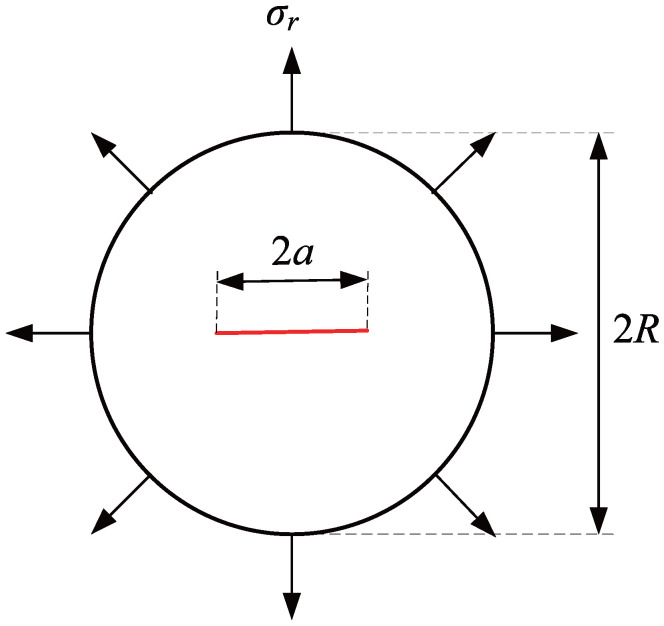
Schematic representation of a circular plate with a central crack subjected to a constant normal traction along the circumference.

To illustrate the capability of the adaptive CT XIGA method based on LR B-splines in accurately representing geometric structures with curved boundaries, this example considers a circular plate with a central crack. The radius of the plate is R=0.1 unit, and the crack length is 2a=0.08 units, passing through the center, as depicted in Figure 11. A constant traction force of $\sigma_{r}=100$ units is applied along the circumferential normal direction. The elastic modulus is $E=3\times 10^{7}$ units, and the Poisson’s ratio is ν=0.25. This example focuses on the analysis of Mode-I SIF, with the theoretical solution referenced in [57]

(20) $$K_{I}=F\sigma_{r}\sqrt{\pi a},$$

(21) $$F=\frac{1-0.5\alpha +1.6873\alpha^{2}-2.671\alpha^{3}+3.2027\alpha^{4}-1.8935\alpha^{5}}{\sqrt{1-\alpha }},$$

where α represents the ratio of the crack length to the diameter of the circular plate and is expressed as α=a/R.

**Figure 12:**
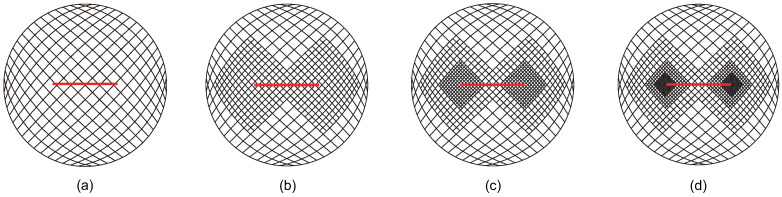
Initial computational mesh (**a**) and the meshes of local refinement at first (**b**), second (**c**), and third (**d**) steps in adaptive CT XIGA.

The adaptive XIGA approach adopts the crack-tip topological refinement strategy and structured mesh refinement strategy to locally refine the mesh for solving the Mode-I SIF in this example. The initial computational mesh, which consists of 16×16 elements, and the first three steps of refined meshes are presented in Figure 12. The geometry of the circular plate is accurately modeled by adaptive CT XIGA based on LR B-splines. As expected, local refinement primarily occurs near the two crack tips. As the number of refinement steps increases, the local refinement regions near the two crack tips become progressively smaller and the element sizes in the vicinity of the two crack tips continue to decrease.

**Figure 13:**
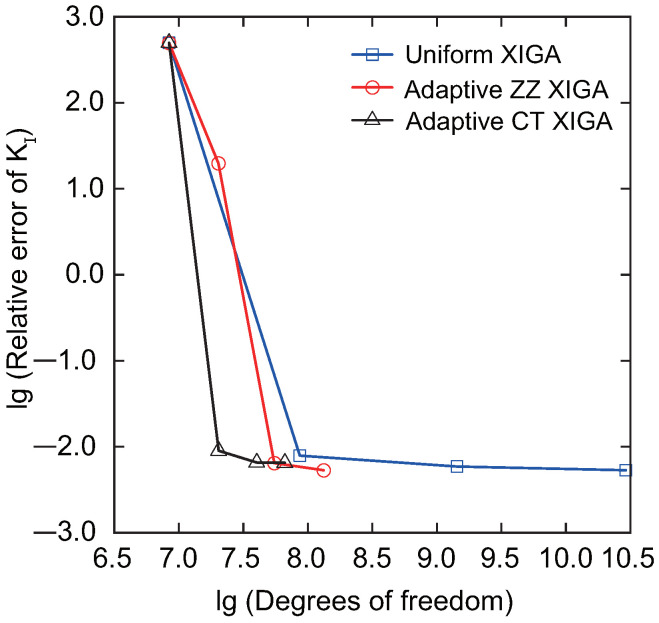
Convergence of the relative error of the Mode-I SIF as a function of the number of DOFs.

**Figure 14:**
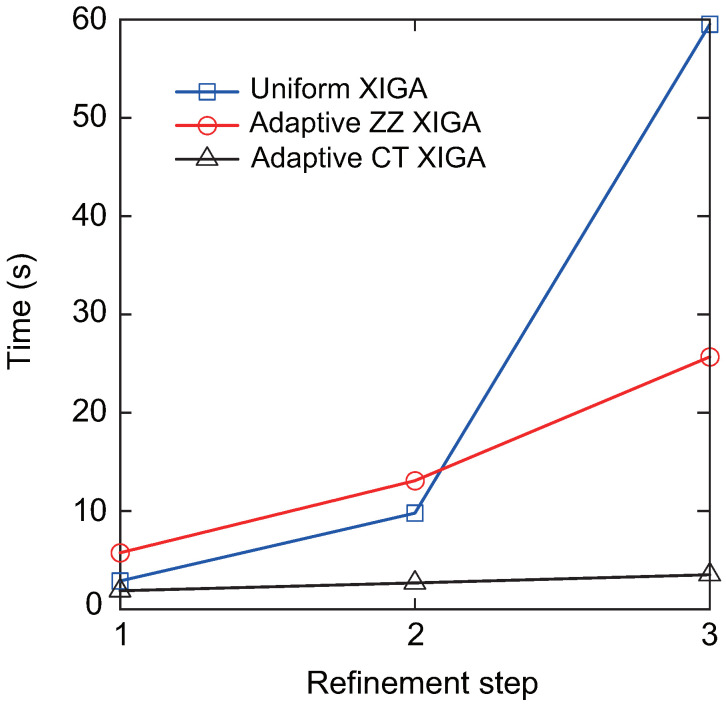
Comparison of computation times as a function of the number of refinement steps.

The convergence curve of the relative error of the Mode-I SIF versus the number of DOFs obtained using adaptive CT XIGA is presented in Figure 13, while the convergence curves corresponding to the uniform XIGA and adaptive ZZ XIGA methods [42] are also included for comparison. These results clearly demonstrate the superior performance of adaptive CT XIGA, achieving a significantly lower relative error with fewer DOFs compared to the other approaches. Figure 14 illustrates the computational time required to solve the SIFs for the two crack tips at different refinement steps using three methods: uniform XIGA, adaptive ZZ XIGA, and adaptive CT XIGA. The results reveal that adaptive CT XIGA exhibits a much lower computational cost compared to uniform XIGA and adaptive ZZ XIGA, particularly with increasing refinement steps. This underscores the superior efficiency of adaptive CT XIGA in terms of computational performance.

**Figure 15:**
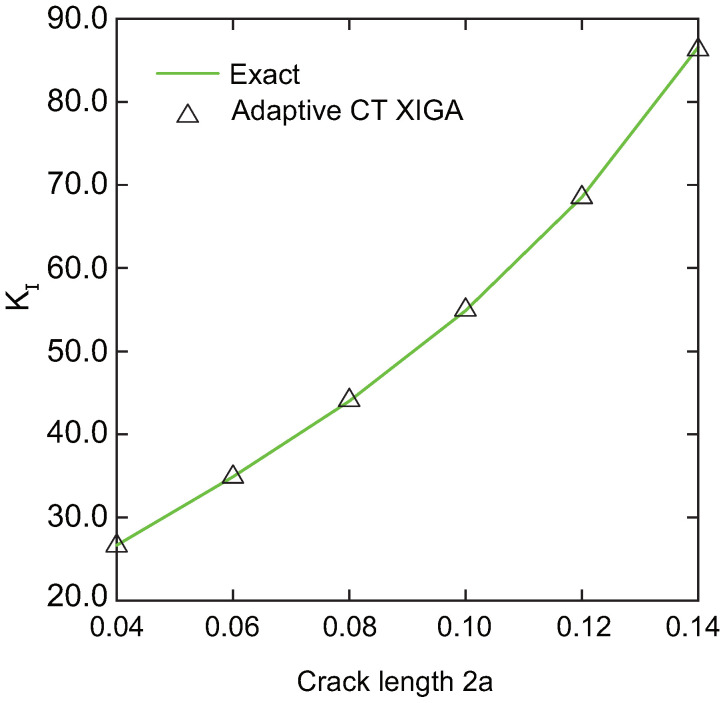
Comparison of Mode-I SIFs for different crack lengths 2a.

To assess the performance of adaptive CT XIGA in addressing the Mode-I SIF, Mode-I SIFs derived from adaptive CT XIGA are compared with exact solutions for various crack lengths, specifically 2a = 0.04 m, 0.06 m, 0.08 m, 0.10 m, 0.12 m, and 0.14 m, as illustrated in Figure 15. There is remarkable agreement between the computed values and their exact solutions, thereby demonstrating that adaptive CT XIGA exhibits efficiency in resolving fracture problems.

### 3.3. Square Plate with a Center Curved Crack

**Figure 16:**
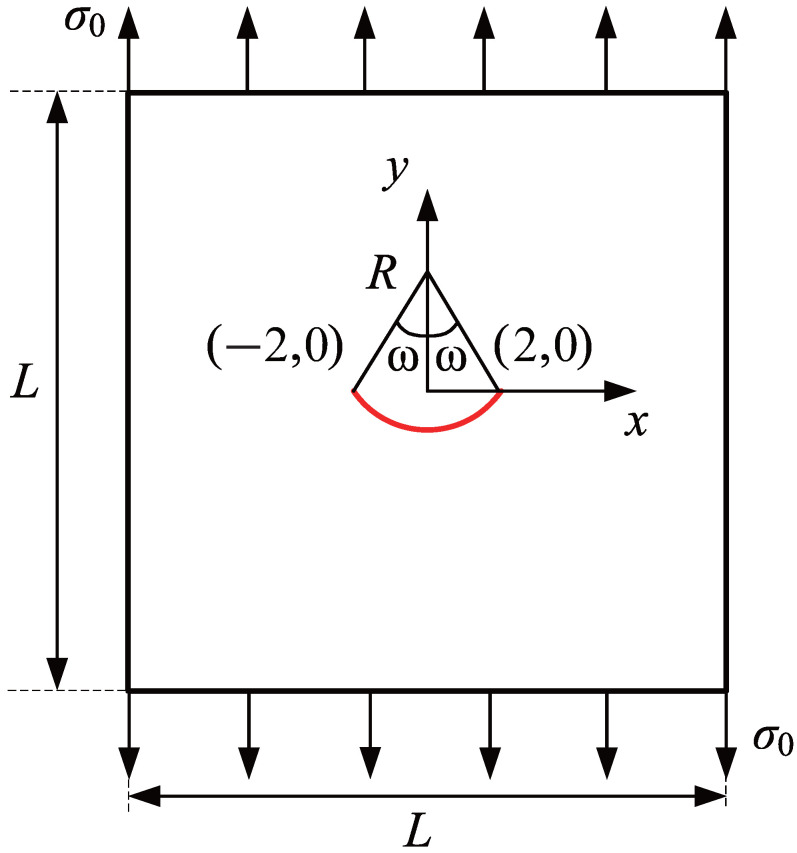
Schematic representation of a square plate with a central curved crack under uniaxial tension.

To demonstrate the capability of the proposed method for simulating both curved cracks and small cracks, a square plate with a side length of L=40 units containing a central curved crack is analyzed, as depicted in Figure 16. The plate has a large edge-to-crack length ratio (>10), indicating a relatively small crack size compared to the overall model. The central curved crack is modeled as a circular arc with endpoints at (−2,0) and (2,0). The crack geometry is further characterized by a radius R=4.25 units and a subtended angle 2ω. The elastic modulus is specified as $E=3\times 10^{7}$ units, while the Poisson’s ratio is set to ν=0.25. The top and bottom edges of the plate are subjected to uniform tensile stress along the y-direction with a magnitude of $\sigma_{0}=1$ unit. For the central curved crack in an infinite plate, the analytical solutions for the mixed-mode SIFs are provided by [42]

(22) $$\begin{array}{cc} K_{I} & =\frac{\sigma_{0}}{2}{\left(\pi R\sin\left(\omega \right)\right)}^{1/2}\left(K\cos\left(\omega /2\right)+\cos\left(3\omega /2\right)\right),\\ K_{II} & =\frac{\sigma_{0}}{2}{\left(\pi R\sin\left(\omega \right)\right)}^{1/2}\left(K\sin\left(\omega /2\right)+\sin\left(3\omega /2\right)\right), \end{array}$$

where $K=\left(1-\sin^{2}\left(\omega /2\right)\cos^{2}\left(\omega /2\right)\right)/\left(1+\sin^{2}\left(\omega /2\right)\right)$.

**Figure 17:**
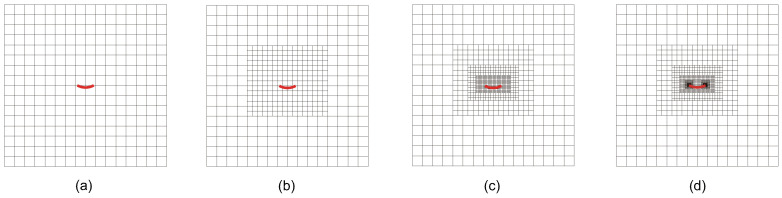
Initial computational mesh (**a**) and the meshes of local refinement at first (**b**), third (**c**), and fifth (**d**) steps in adaptive CT XIGA.

The convergence behavior for the mixed-mode SIFs is studied by setting ω=28.0725. The initial computational mesh, consisting of 16×16 elements, is presented in Figure 17, along with the locally refined meshes of adaptive CT XIGA at the first, third, and fifth refinement steps. Due to the small size of the curved crack, the local refinements during the first three steps are focused around the crack face, while the fourth and fifth refinement steps predominantly occur near the crack tips.

**Figure 18:**
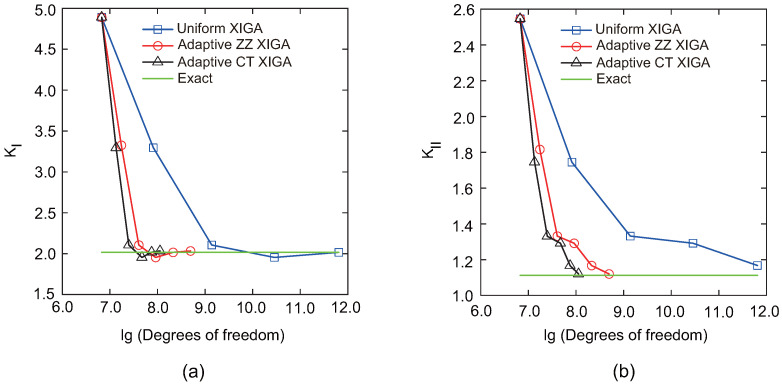
Convergence of the mixed-mode SIFs as a function of the number of DOFs for the square plate with a center curved crack: $K_{I}$ (**a**) and $K_{II}$ (**b**).

**Figure 19:**
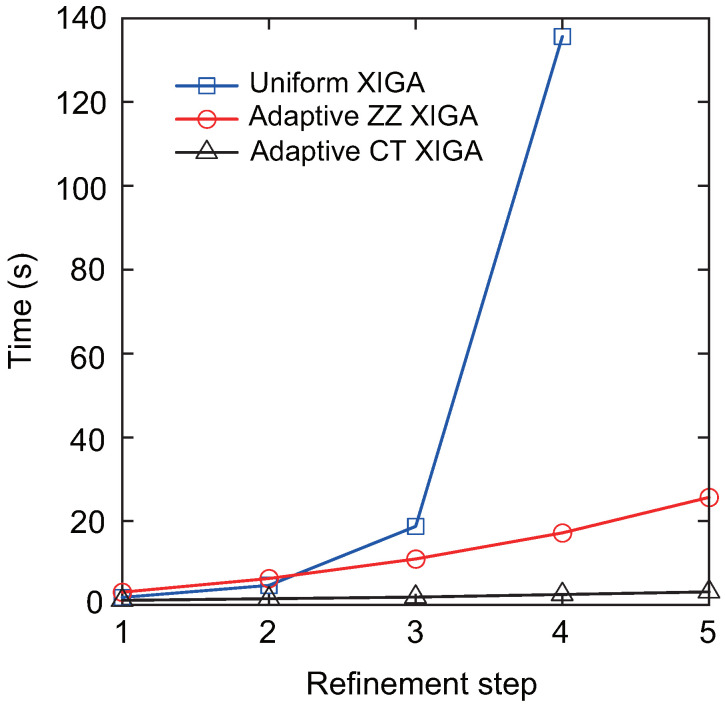
Comparison of computation times as a function of the number of refinement steps.

Figure 18 illustrates the convergence behavior of the mixed-mode SIFs, $K_{I}$ and $K_{II}$, with respect to the degrees of freedom. The results achieved with adaptive CT XIGA are compared with those from uniform XIGA and adaptive ZZ XIGA [42]. All the methods demonstrate convergence in computing the mixed-mode SIFs. Specifically, the Mode-I SIF $K_{I}$ converges after the fourth mesh refinement, whereas the Mode-II SIF $K_{II}$ requires five mesh refinements to converge. Among the three methods, adaptive CT XIGA achieves the fastest convergence rate. When calculating the mixed-mode SIFs using the interaction integral method, sufficiently small mesh sizes near the crack tips are crucial, particularly for small cracks. While all three methods—adaptive ZZ XIGA, uniform XIGA, and adaptive CT XIGA—can adequately refine the mesh near the crack tips, their computational time requirements differ significantly. Figure 19 illustrates the computational time required by each method at different refinement steps to calculate the mixed-mode SIFs. For four refinement steps, uniform XIGA requires substantially more computational time than adaptive ZZ XIGA and adaptive CT XIGA. Moreover, adaptive CT XIGA shows much lower computational time and a slower increase in computational time per refinement step compared to the other two methods. This highlights the advantage of adaptive CT XIGA in terms of computational efficiency when analyzing small cracks.

**Figure 20:**
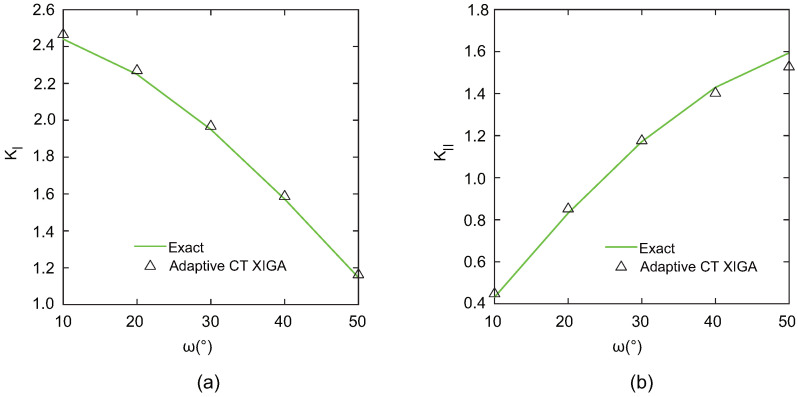
Comparison of the mixed-mode SIFs at different values of ω: $K_{I}$ (**a**) and $K_{II}$ (**b**).

To evaluate the performance of adaptive CT XIGA in modeling curved cracks, the mixed-mode SIFs are investigated for various values of ω. As ω increases, the Mode-I SIF ($K_{I}$) exhibits a decreasing trend, whereas the Mode-II SIF ($K_{II}$) shows a corresponding increase. The results computed by adaptive CT XIGA with five local refinements for the mixed-mode SIFs are in very good agreement with the exact solutions, as shown in Figure 20. This agreement highlights the robustness and accuracy of adaptive CT XIGA in resolving the mixed-mode SIFs associated with a curved crack.

### 3.4. Cantilever Beam with an Edge Crack

**Figure 21:**
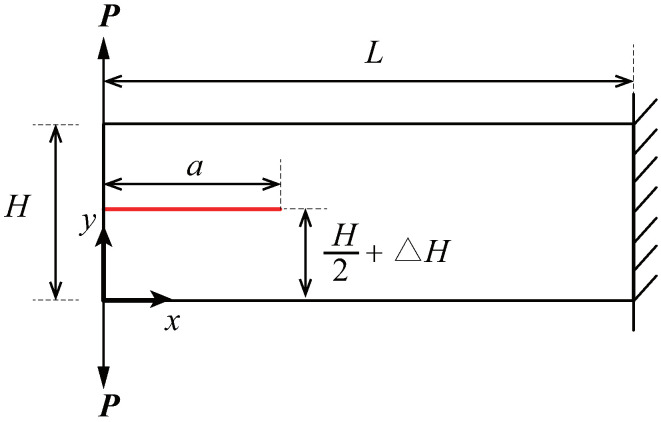
Schematic representation and loading conditions of a cantilever beam with an edge crack.

To demonstrate the applicability of adaptive CT XIGA to crack propagation simulation, this example investigates the propagation of an edge crack in a cantilever beam. The geometric configuration of the cantilever beam, with dimensions L=6 units and H=2 units, is illustrated in Figure 21. A planar Cartesian coordinate system is defined, with its origin at the bottom-left corner of the beam. An edge crack of length a=2.05 units is positioned slightly above the mid-plane of the left boundary, offset by ΔH=0.02 units. The beam is subjected to concentrated vertical loads of P=1 unit, applied vertically at its top-left and bottom-left corners, while the beam’s right boundary is fixed. The material properties are defined under plane stress conditions, with the elastic modulus of E=100 units and the Poisson’s ratio of ν=0.3.

The adaptive CT XIGA approach based on LR B-splines is employed to simulate edge crack propagation in the cantilever beam. The initial mesh consists of 24×8 uniform elements, and the crack increment length is set to Δa=0.15 units, consistent with Ref. [43]. The total number of crack propagation steps is 10. According to Ref. [43], to ensure accuracy in the crack propagation path, the interaction integral radius $r_{J}=3\sqrt{S_{tip}}$ should be smaller than the crack increment length Δa. Therefore, adaptive CT XIGA requires at least three local refinement steps.

**Figure 22:**
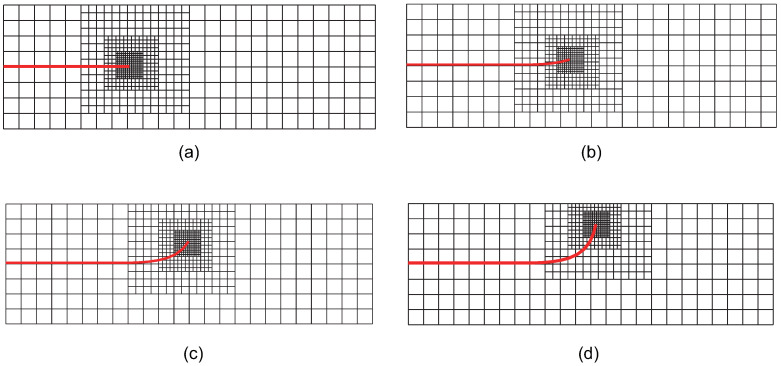
The locally refined meshes of adaptive CT XIGA applying three local refinement steps for crack growth at step 0 (**a**), step 4 (**b**), step 7 (**c**), and step 9 (**d**).

Figure 22 illustrates the locally refined meshes during crack propagation at step 0, step 4, step 7, and step 9. The figure shows that, as the crack propagates, local mesh refinement primarily occurs in the vicinity of the crack tip, ensuring that the element size near the crack tip remains sufficiently small. Starting from step 4 (Figure 22b), the crack propagation direction begins to deviate from its initial trajectory, extending upward from the horizontal axis. This behavior is consistent with the actual propagation scenario, where the initial crack is slightly above the centerline of the cantilever beam and is subjected to concentrated loading. By step 7 (Figure 22c), the deviation of the crack propagation direction from its initial trajectory becomes more pronounced. Additionally, at step 9 (Figure 22d), the crack has propagated near the model boundary, where further propagation and refinement are significantly affected by boundary effects. This is one reason that the number of propagation steps is limited to 10, with no further extension considered.

**Figure 23:**
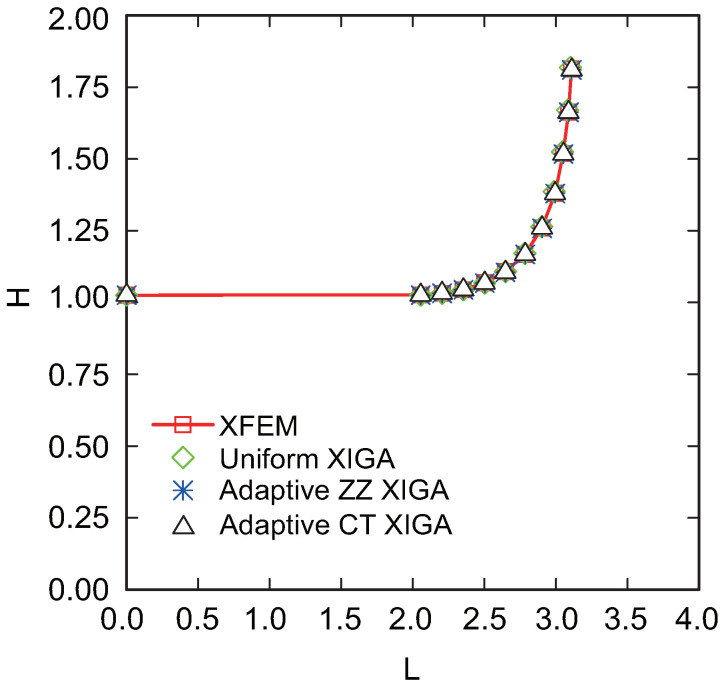
Comparison of crack growth paths of the cantilever beam with an edge crack.

**Figure 24:**
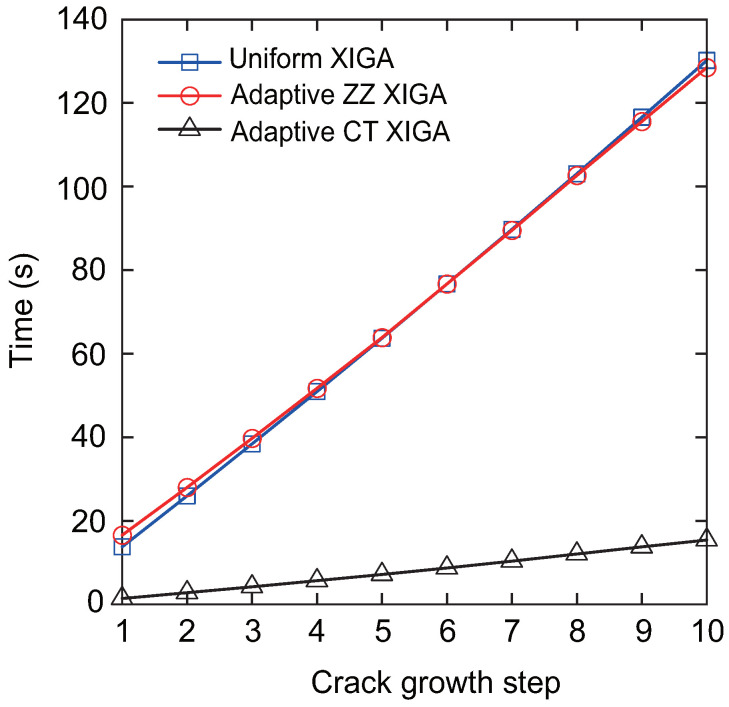
Comparison of computation times as a function of the number of crack growth steps.

Figure 23 presents the crack propagation paths achieved via uniform XIGA, adaptive ZZ XIGA, and adaptive CT XIGA with three refinement steps, alongside the crack propagation path computed by the XFEM with 4800 elements [58]. The close agreement among the crack propagation paths generated by these methods highlights the effectiveness of adaptive CT XIGA in accurately simulating edge crack propagation. However, as illustrated in Figure 24, the computational time required to simulate crack propagation differs significantly among these methods.

Figure 24 shows the computational time for each crack propagation step using uniform XIGA, adaptive ZZ XIGA, and adaptive CT XIGA with three refinement steps. The computational times for uniform XIGA and adaptive ZZ XIGA are comparable. In contrast, adaptive CT XIGA requires substantially less computational time per propagation step than both uniform XIGA and adaptive ZZ XIGA. The primary reason for this difference is attributed to the computational time required for SIF evaluation. Adaptive CT XIGA significantly reduces the computational cost of SIF evaluation compared to uniform XIGA and adaptive ZZ XIGA, as shown in earlier examples. The smaller the crack increment length Δa, the smoother the crack propagation path becomes. However, since the crack increment length Δa must be greater than the interaction integral radius $r_{J}=3\sqrt{S_{tip}}$, the element size near the crack tip must be sufficiently small. If fine elements are used throughout the entire domain, as is the case with uniform XIGA, the computational cost increases substantially, especially when the crack size is relatively small compared to the overall model or when the crack increment length is set to a small value. A more computationally efficient approach involves using fine elements near the crack tip and coarser elements in regions farther from the crack tip, as implemented in adaptive ZZ XIGA and adaptive CT XIGA.

However, adaptive ZZ XIGA requires multiple recovery steps and displacement variable computations to calculate the SIFs, leading to substantially higher computational time compared to adaptive CT XIGA. Consequently, adaptive CT XIGA is highly suitable for simulating crack propagation, delivering both superior accuracy and computational efficiency.

### 3.5. Square Plate with a Center-Inclined Crack

**Figure 25:**
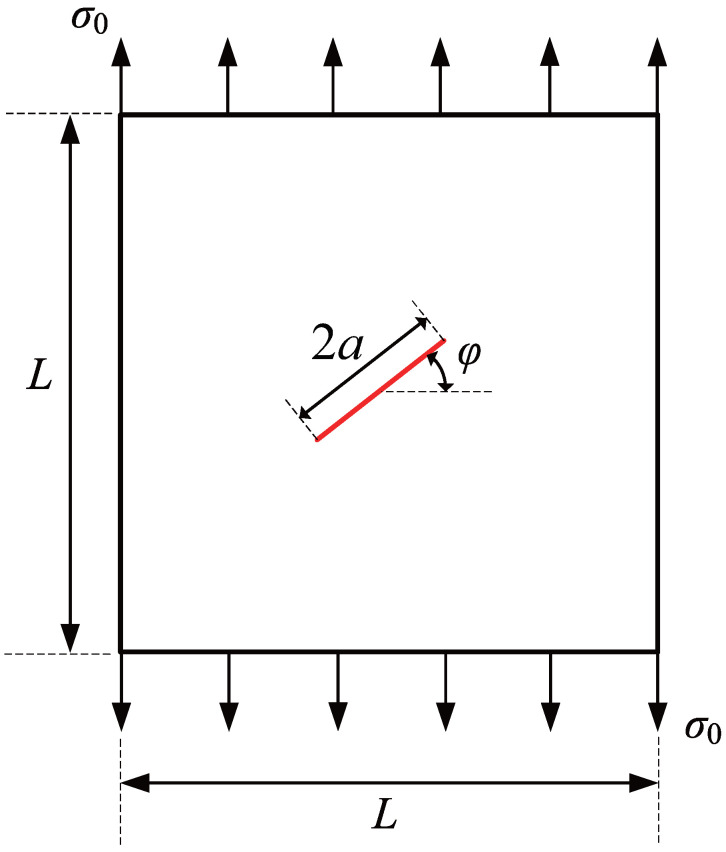
Schematic representation and loading conditions of a square plate with a center-inclined crack.

To further demonstrate the effectiveness of the adaptive CT XIGA method in simulating crack propagation, this example investigates the propagation of a centrally inclined crack in a square plate under uniaxial tension, as shown in Figure 25. The length of the square plate is L=10 units, and the crack length is 2a=1 unit. The angle of inclination of the crack relative to the horizontal direction is represented by φ. The material parameters are E=1000 units and ν=0.3. A uniaxial uniform tensile stress of $\sigma_{0}=1$ unit is applied to the top and bottom edges of the square plate in the *y*-direction.

**Figure 26:**
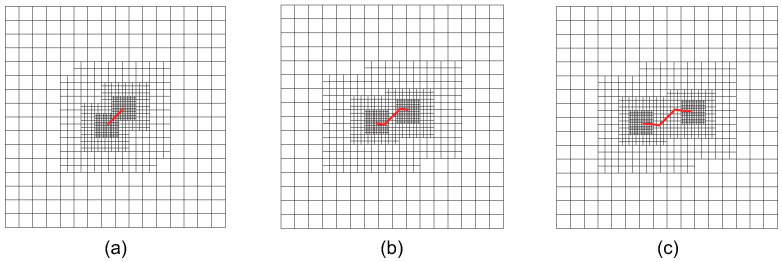
The locally refined meshes of adaptive CT XIGA applying three local refinement steps for crack growth at step 0 (**a**), step 1 (**b**), and step 2 (**c**) when φ=45.

First, the crack inclination angle φ is set to 45, with a crack growth increment Δa of 0.38 units. The crack propagates over a total of three steps for both crack tips, in accordance with Ref. [58]. The initial computational mesh consists of 16×16 elements. To ensure that the interaction integral radius, $r_{J}=3\sqrt{S_{\mathrm{tip}}}$, remains smaller than the crack growth increment Δa, at least three local refinement steps are required for adaptive CT XIGA. The meshes generated by adaptive CT XIGA during the first three crack growth steps are presented in Figure 26. As the crack propagates, local mesh refinement consistently occurs in the vicinity of the two crack tips.

**Figure 27:**
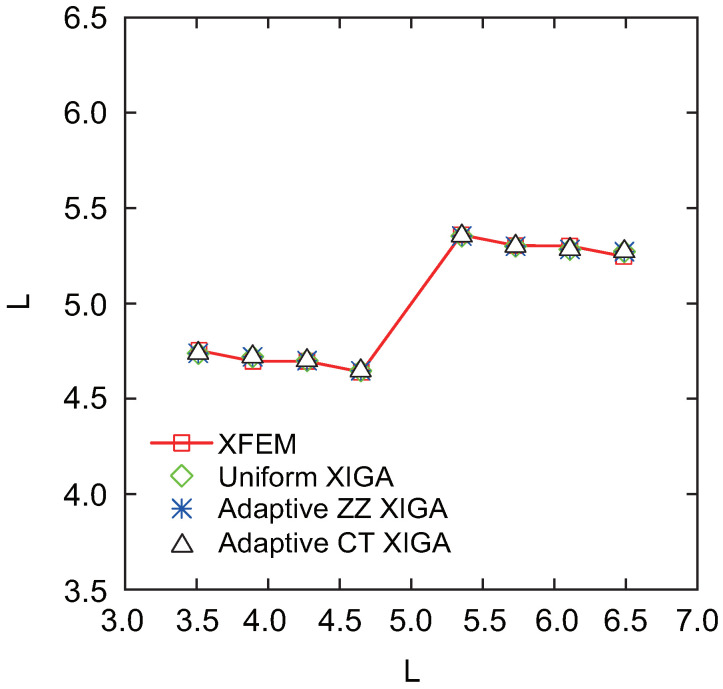
Comparison of crack growth paths of the square plate with a center-inclined crack when φ=45.

**Figure 28:**
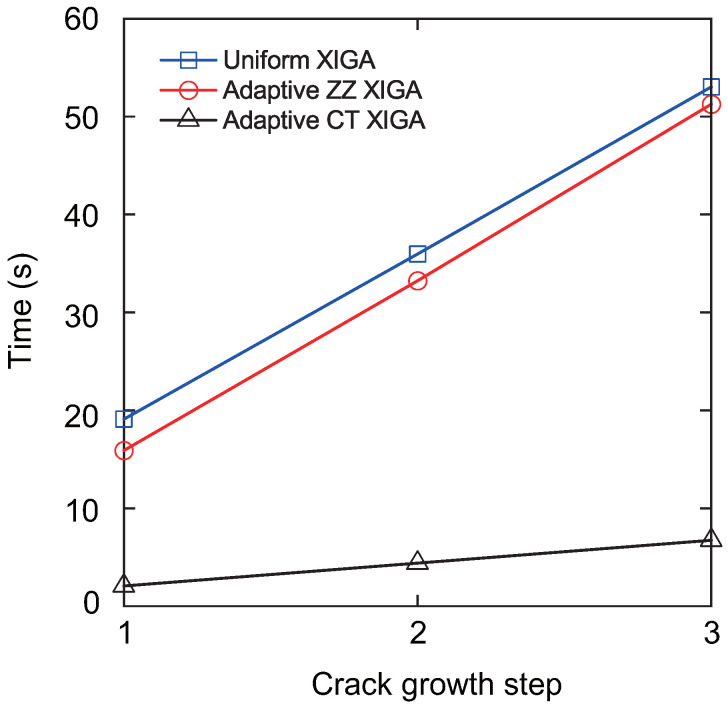
Comparison of computation times as a function of the number of crack growth steps.

Figure 27 presents the crack growth paths obtained using uniform XIGA, adaptive ZZ XIGA, adaptive CT XIGA with three refinement steps, and the XFEM method [58]. The results demonstrate strong agreement between the crack growth paths produced by these methods, highlighting the effectiveness and accuracy of adaptive CT XIGA in simulating inclined crack propagation. A comparison of the computation times for each crack propagation step using uniform XIGA, adaptive ZZ XIGA, and adaptive CT XIGA with three refinement steps is illustrated in Figure 28. It is evident that both the time required for each propagation step and the time increase rate indicate that adaptive CT XIGA outperforms the other methods.

**Figure 29:**
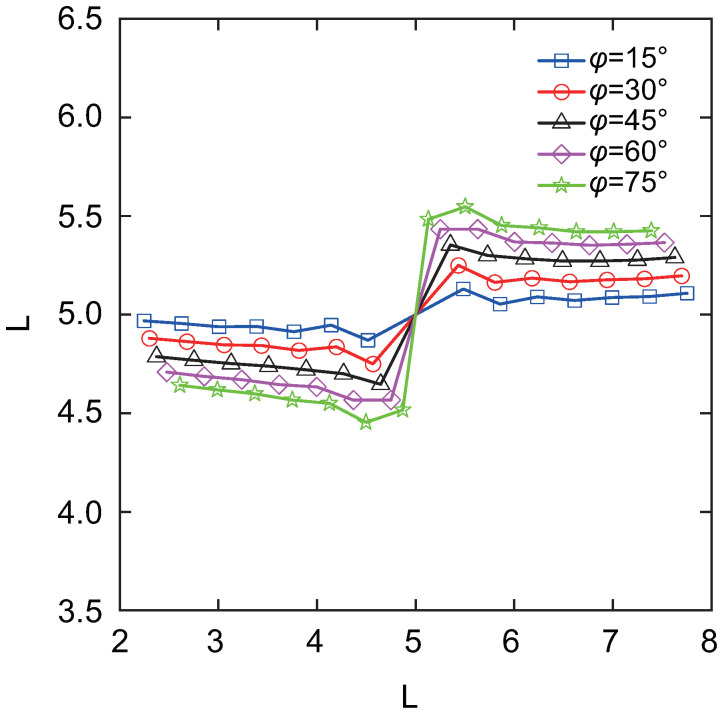
Crack growth paths of the center-inclined crack in the square plate by adaptive CT XIGA using three local refinement steps for different crack inclination angles.

Then, the crack growth increment remains unchanged, and the total number of crack propagation steps for both tips is set to 6. The crack propagation paths for different crack inclination angles, obtained using adaptive CT XIGA with three refinement steps, are shown in Figure 29. For each crack inclination angle, the crack tip propagation directions are symmetric, and, in all cases, the cracks ultimately propagate in the horizontal direction.

### 3.6. Square Plate with Two Edge Cracks

**Figure 30:**
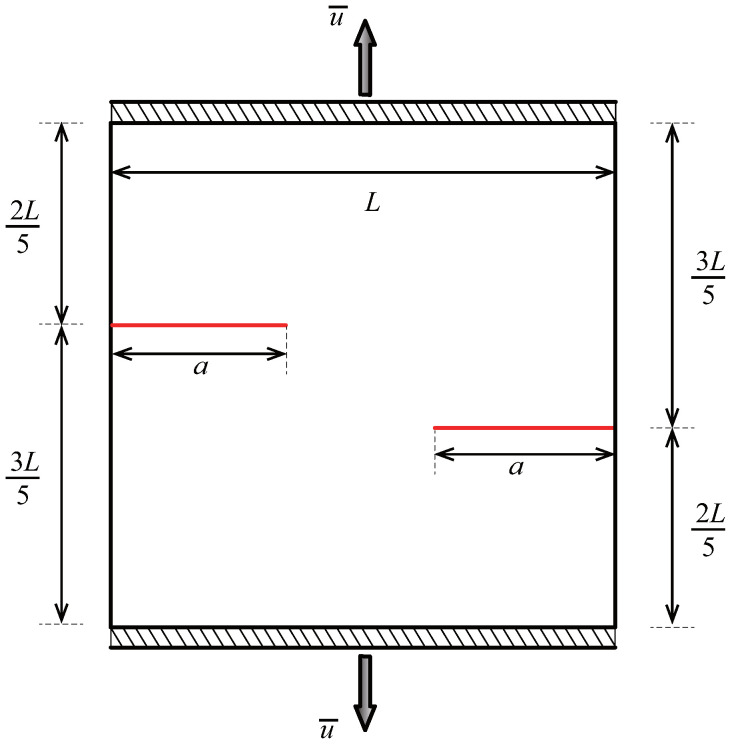
Schematic representation and loading conditions of a square plate with two edge cracks.

To evaluate the proposed method’s effectiveness in simulating multi-crack propagation, the final numerical example analyzes a representative example of double-crack propagation. Figure 30 illustrates a square plate with a side length of L=10 units. The plate features two edge cracks of length a=3.5 units, positioned symmetrically on the left and right edges. The material properties of the plate are defined by the elastic modulus E=1 unit and the Poisson’s ratio ν=0.25. Vertical displacements, $\bar{u}=0.01$ unit, are applied along both the top and bottom boundaries of the plate.

**Figure 31:**
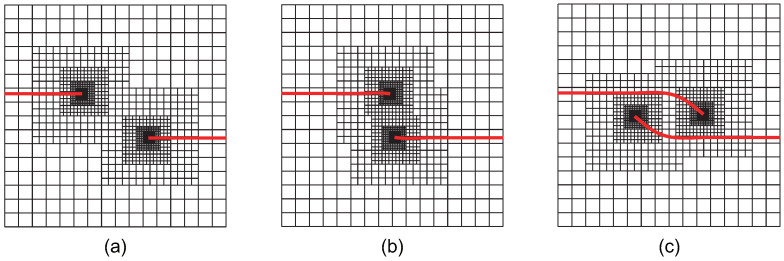
The locally refined meshes of adaptive CT XIGA applying four local refinement steps for crack growth at step 0 (**a**), step 8 (**b**), and step 17 (**c**).

The initial computational mesh is set to 16×16 elements, and the crack growth increment is selected as Δa=a/20, with a total of 18 crack propagation steps, consistent with Ref. [43]. To ensure that the interaction integral radius $r_{J}=3\sqrt{S_{\mathrm{tip}}}$ does not exceed the crack growth increment Δa, adaptive CT XIGA requires at least four local refinement steps. Figure 31 presents meshes of adaptive CT XIGA for the crack growth at the initial stage, the 8th step, and the 17th step. As expected, with crack propagation, local mesh refinement predominantly occurs near the two crack tips, ensuring sufficiently small element sizes.

**Figure 32:**
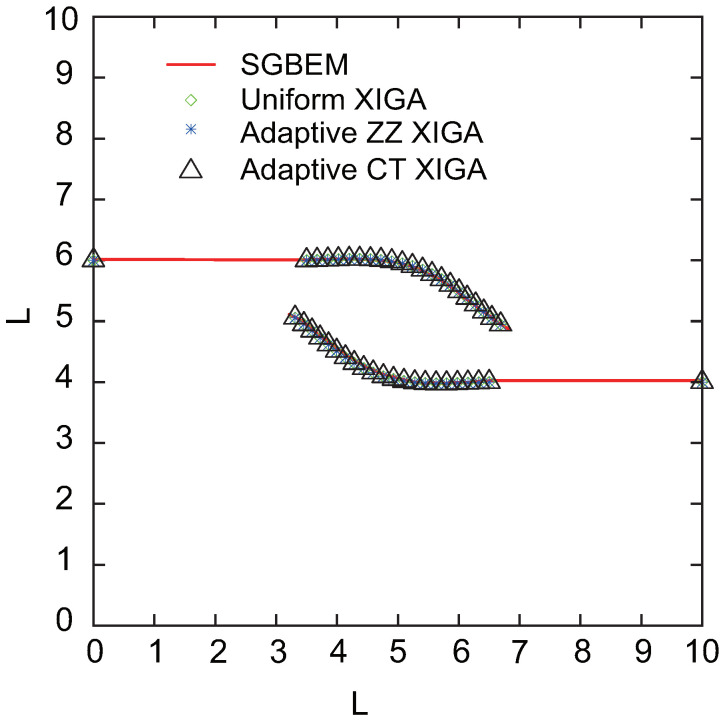
Comparison of crack growth paths of the square plate with two edge cracks.

**Figure 33:**
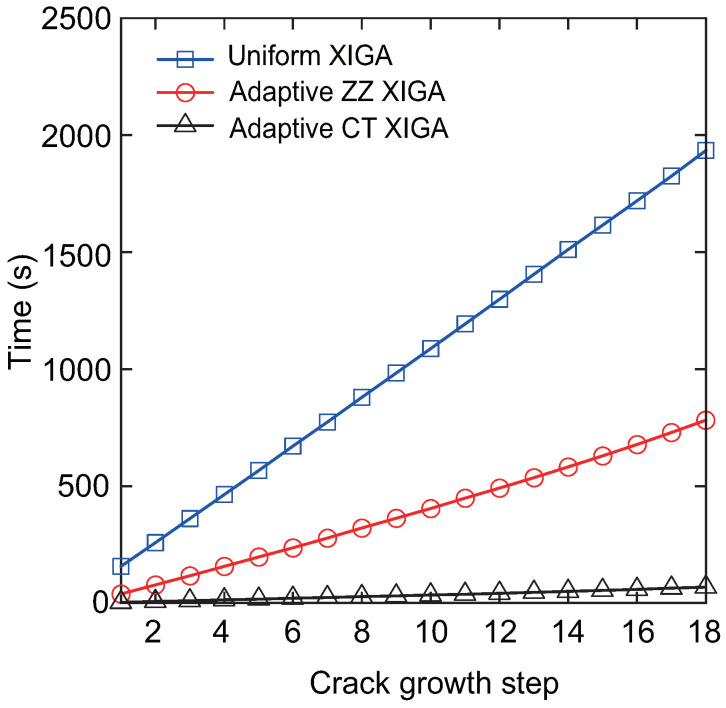
Comparison of computation times as a function of the number of crack growth steps.

The crack growth paths simulated using uniform XIGA, adaptive ZZ XIGA, adaptive CT XIGA with four refinement steps, and the SGBEM method [59] are presented in Figure 32. The results indicate that the crack growth paths obtained by these methods show strong agreement, highlighting the effectiveness and accuracy of adaptive CT XIGA in simulating multiple-crack propagation. Similar to the single-crack propagation simulation, the computational time for uniform XIGA, adaptive ZZ XIGA, and adaptive CT XIGA differs significantly, as illustrated in Figure 33. Because of the use of four refinement steps, uniform XIGA involves a large number of elements (up to 65536), resulting in significantly higher computational time per propagation step than adaptive ZZ XIGA and adaptive CT XIGA. Furthermore, compared to adaptive ZZ XIGA, adaptive CT XIGA requires significantly less time per propagation step and for the entire 18 steps. This highlights the significant computational efficiency advantage of adaptive CT XIGA in simulating multiple-crack propagation.

## 4. Conclusions

In this study, an adaptive CT XIGA framework based on LR B-splines has been presented for efficient and accurate fracture modeling in two-dimensional solids. By utilizing localized enrichment functions, the proposed method enables the representation of cracks without requiring the computational mesh to conform to the crack geometry, thereby eliminating the need for remeshing during crack propagation. The integration of LR B-splines, which offer high-order continuity and flexible local refinement capabilities, greatly enhances both the computational accuracy and efficiency of fracture simulations. Several local mesh refinement strategies have been designed and developed based on the positions of cracks and crack tips. The error convergence rates and computational times of different refinement strategies have been systematically compared. Among these, the crack-tip topological refinement strategy has been identified as the most effective strategy due to its ability to achieve the highest convergence rate while demonstrating the lowest computational cost. Numerical examples have been presented to validate the accuracy and effectiveness of the adaptive CT XIGA method in computing SIFs and simulating crack propagation. Furthermore, compared to a-posteriori-error-based adaptive XIGA, the adaptive CT XIGA approach exhibits superior error convergence rates and achieves substantial reductions in computational time, making it especially advantageous for crack propagation simulations. In addition, the crack-tip topological refinement strategy offers a simpler and more streamlined implementation of local mesh refinement relative to a-posteriori-error-based methods. By integrating the geometric precision of isogeometric analysis with concepts inspired by the XFEM and leveraging the efficiency of local refinement, the proposed method offers a robust and computationally efficient framework for addressing complex fracture problems in solid mechanics. It has potential applications in crack growth modeling for plates, shells, and three-dimensional structures.

## Data Availability

The original contributions presented in the study are included in the article, further inquiries can be directed to the corresponding authors.
